# A Case Report of Non-secretory Multiple Myeloma

**DOI:** 10.7759/cureus.29571

**Published:** 2022-09-25

**Authors:** Maria A Kelley, Andrea Mestre, Maria F Ayau, Arpit Arora, Ratesh Khillan

**Affiliations:** 1 General Medicine, Universidad de Guayaquil, Guayaquil, ECU; 2 General Practice, Universidad del Rosario, Bogota, COL; 3 General Practice, Universidad Francisco Marroquín, Guatemala City, GTM; 4 Medicine and Surgery, Dayanand Medical College and Hospital, Ludhiana, IND; 5 Hematology / Oncology, Kingsbrook Jewish Medical Center, New York, USA

**Keywords:** immunoglobulin, monoclonal gammopathy, malignancy, non secretory, multiple myeloma

## Abstract

Non-secretory multiple myeloma (NSMM) is a hematological malignancy that presents as a unique clinical form of multiple myeloma with proliferation of plasmacytic cells that cannot secrete or synthesize immunoglobulins. Its prevalence as a hematologic malignancy in adults is low. Our article describes a case of a 68-year-old lady presenting with diffuse scattered lytic lesions throughout the axial and visualized appendicular skeleton without lymph node involvement. She was treated with chemotherapy with a good clinical response. We will present a case report to showcase the differences in pathophysiology, clinical features, diagnosis, and management compared to secretory multiple myeloma.

## Introduction

Multiple myeloma (MM) represents 1-2% of all cancers and 17% of hematologic malignancies, with an incidence of 34,000 new cases per year in the USA and 180,000 new cases per year worldwide [1]. Multiple myeloma is largely a disease of older adults, of which the median age of diagnosis is 65 to 74 years of age, being twice as common in African-Americans as compared to Caucasians and lowest among the Chinese and Japanese [1]. Non-secretory multiple myeloma, a myeloma subtype with evolving diagnostic criteria, accounts for 2-4% of all cases of multiple myeloma [1]. Because of how rare this variant is, it usually poses a diagnostic challenge to clinicians [2]. 

Multiple myeloma is a hematological malignancy with plasmacytic proliferation of the bone marrow secreting immunoglobulins leading to gammopathy along with CRAB features (hypercalcemia, renal insufficiency, anemia and bone lesions) [2]. The proliferation of this monoclonal (M) protein is characterized by the presence of heavy and light chains or just by light chains, which can also be found in the urine [2]. Non-secretory myeloma is a rare myeloma subtype with more than 10% of monoclonal plasma cells in the bone marrow; it shows evidence of damage in end-organs or a biopsy that proves the presence of a plasmacytoma, as well as negative results on serum, urine electrophoresis and immunofixation studies due to the fact that they cannot secrete immunoglobulins such as IgG, IgA, IgM, Kappa and Lambda [3].

Therefore, the aim of this case report is to provide a better understanding about non-secretory multiple myeloma and to review the known pathophysiologic basis of the non-secretion nature and its implications for diagnosis, treatment, and prognosis in the clinic [4]. 

## Case presentation

Our patient is a 68-year-old African-American female with a past medical history of mitral valve disease, hypertension, and hysterectomy secondary to fibroids, who complained of shoulder pain initially involving the left shoulder and later the right shoulder since two months prior. The patient's family history was remarkable for a father with prostate cancer and a brother with colon cancer. Upon examination, there was a decreased range of motion in both shoulders and tenderness to the touch over the left proximal humerus. No lymphadenopathy was noted on the clinical exam. 

Blood analysis was taken and showed no abnormalities (Table 1); serum protein electrophoresis (SPEP), serum immunofixation, and serum free light chain (sFLC) assay were taken and exhibited normal results (Table 2, 4, 5). Serum immunoglobulin A, G, and M results were normal (Table 3). X-rays showed multiple pathological fractures and lytic lesions involving the left humerus, lumbar spine, and left ninth rib. A CT scan of the abdomen and pelvis showed diffuse lytic lesions. The patient was surgically intervened with pinning of the right and left humerus and right and left femur as a prophylaxis measure for future pathological fractures. A week later the patient underwent a bone marrow biopsy, flow cytometry, and fluorescence in situ hybridization (FISH) study. The bone marrow biopsy showed hypercellularity (70%) for age composed of trilinear maturing hematopoiesis with subtotal replacement of marrow by sheets of plasma cells comprising 80% of the cellularity. Flow cytometry done on the bone marrow aspirate showed neoplastic plasma cells with the following phenotype: CD45-, CD19-, CD20-, CD38+, CD56+, CD138+, and clg kappa +. The FISH study done on the bone marrow aspirate showed a gain of chromosomes 7, 9, 15, and 1q21, as well as monosomy of chromosome 13 without any loss of Tumoral Protein (TP) 53 or the presence of translocation Cyclin D1 (CCND1)/Immunoglobulin Heavy Chain (IGH) and Fibroblast Growth Factor 3 (FGFR3)/IGH. The patient underwent a palliative and consolidation course of external radiation therapy to both the proximal humerus and was actively treated with a plan of bortezomib 1.96 mg/m2 three weeks on and one week off.

**Table 1:** Patient´s blood analysis RBC=red blood cell count; MCV=mean corpuscular volume; MCH=mean corpuscular hemoglobin; MCHC=mean corpuscular hemoglobin concentration; RDW=red blood cell distribution width; eGFR=estimated glomerular filtration rate

Blood analysis first week	Patient’s results	Normal range
While blood cells	5.2	3.1-10.6 10^3/uL
RBC	3.60	3.65-4.69 10^6/uL
Hemoglobin	11.6	10.8-14.2 g/dL
Hematocrit	32.7	37.7-53.7 %
MCV	90.9	77.0-97.0 fL
MCH	32.3	27.0-31.2 pg
MCHC	35.5	31.8-35.4 g/dL
RDW	14.8	10.2-14.6
Platelet count	226	121-412 10^3/uL
Sodium	140	132-146 mEq/L
Potassium	3.8	3.5-5.5 mEq/L
Creatinine	0.66	0.60-1.20 mg/dL
Glucose	87	51-105 mg/dL
Calcium	9.6	8.6-10.3 mg/dL
Total protein	6.3	6.0-8.3
Albumin	4.12	3.5-5.2
Alkaline Phosphatase	57	34-104 U/L
eGFR	103	60-2000 mL/min/1.73m2

**Table 2:** Serum protein electrophoresis results SPEP=Serum protein electrophoresis

SPEP	Patient’s results	Normal range
Total protein	6.5	6.0-8-3 g/dL
Alpha 1 Concentration	0.26	0.10-0.40 g/dL
Alpha 2 Concentration	0.75	0.40-1.20 g/dL
Beta 1 Concentration	0.36	0.60-1.30 g/dL
Gamma Concentration	1.10	0.50-1.60 g/dL
M-SPIKE	None detected	

**Table 3:** Patient´s immunoglobulin A, G, and M results

	Patient’s results	Normal range
Immunoglobulin A	72	70-320 mg/dL
Immunoglobulin G	1269	600-1741 mg/dL
Immunoglobulin M	31	50-300 mg/dL

**Table 4:** Patient´s serum immunofixation result

	Patient’s results	Comments
Serum Immunofixation	Normal	Normal polyclonal immunofixation pattern

**Table 5:** Serum free light chain (sFLC) assay results

	Patient’s results	Normal range
Kappa Free Light Chain	1.46	0.33-1.94 mg/dL
Lambda Free Light Chain	0.92	0.57-2.63 mg/dL
Kappa/Lambda Ratio	1.59 H	0.26-1.65 mg/dL

## Discussion

MM is a unique varying disease of the plasma cells [5]. It presents with an unchecked growth of monoclonal plasma cells in the bone marrow that is accompanied by the production of dysfunctional immunoglobulin chains [5]. A small number of patients with multiple myeloma can be characterized by the absence of monoclonal proteins in serum and urine, categorizing it as non-secretory multiple myeloma (NSMM), and in 85% of these patients, the presence of cytoplasmic M-proteins within plasma cells have been demonstrated indicating immunoglobulin synthesis [6].

Although the exact pathophysiology of non-secretory multiple myeloma is not completely understood, some studies have found that some NSMM cells secrete Ig, but then become trapped in the cytoplasm and quickly degrade (4). The presentation of NSMM is similar to multiple myeloma except for renal disease; the principal manifestations include lytic bone lesions (as seen in our patient), anemia, and hypercalcemia (CRAB findings) [7]. Our patient was complaining of two months of pain initially in the left shoulder followed by pain in the right shoulder. X-ray findings were consistent with multiple pathological fractures (humerus, lumbar spine, and left ninth rib). 

The workup for diagnosis of NSMM follows the same guidelines as for patients with secretory MM. Primary laboratory studies for NSMM should incorporate complete blood count (CBC), serum protein electrophoresis, immunofixation electrophoresis (IFE), sFLC assay, and quantitative Ig levels [8]. Cases with suspected NSMM need a bone marrow biopsy for cytology, histopathology, flow cytometry CD138, and FISH [9]. The diagnosis of NSMM is assessed by clinical manifestations, normal serum and urine protein electrophoresis, immuno-electrophoresis, and the presence of 10% of monoclonal bone marrow plasma cells (9). Our patient met the criteria for the diagnosis of NSMM, which according to the International Myeloma Working Group is defined by the absence of M protein in serum or urine, bone marrow plasmacytosis, and related organ or tissue impairment [10]; This patient also had a biopsy of the bone marrow that revealed 85% of plasma cells. Cytogenetic analysis showed a gain of chromosomes 7, 9, 15, and 1q21, and monosomy of chromosome 13. At flow cytometry of the bone marrow, the patient had the following phenotype: CD138+, CD 56+, CD38+, which characterized the patient as having a good prognosis [9]. There was no loss of TP53 or presence of translocation of CCND1/IGH and FGFR3/IGH. 

Considering the low incidence of NSMM along with the ineligibility of patients for clinical trials, the exact clinical nature and prognosis of the disease are not fully understood [4]. There is not sufficient research that suggests the NSMM treatment differs from the secreting MM. The treatment will depend on the outcome of the patient, general condition, and eligibility for hematopoietic stem cell transplantation (HSCT) [11]. 

However, monitoring the response of the patient is where it differs from MM, mainly because clinicians do not have the option to use serum and urine Ig studies as a measure of tumor burden [4]. Serial bone marrow studies provide direct examination and therefore are qualified as the gold standard; however, the time, cost, and patient discomfort make them non-viable options from a practical point of view. As a consequence, the focus turns to imaging studies such as PET/CT scans [4]. Since NSMM is a very rare disease, the clinical course and prognosis are not completely distinguished. According to the current research available, NSMM appears to be less threatening than secretory MM [4]. 

Our patient was diagnosed with NSMM four years ago, and she reported no complaints during her last follow-up visit. She continues with bortezomib therapy and yearly skeletal reviews.

## Conclusions

Non-secretory multiple myeloma is a rare type of multiple myeloma with monoclonal plasmocytic proliferation of the bone marrow that cannot secrete or synthesize immunoglobulins. Its prevalence as a hematologic malignancy is low, posing a diagnostic challenge to clinicians. A careful initial evaluation is crucial to collecting the correct information, which is necessary in order to define a long-term strategy for disease management and assessment. Monitoring non-secretory multiple myeloma is based primarily on imaging studies such as PET/CT scans and frequent bone marrow samplings. More studies need to better define the course and the outcome of this entity.
